# The expected prisoner’s dilemma – With rationally arising cooperation

**DOI:** 10.1371/journal.pone.0239299

**Published:** 2020-09-30

**Authors:** Richard J. Arend

**Affiliations:** School of Business, College of Management & Human Service, University of Southern Maine, Portland, ME, United States of America; Peking University, CHINA

## Abstract

Currently, there is no satisfying answer to how cooperation arises rationally in a single-play prisoner’s dilemma game with complete information. When player types are known, as well as payoffs and actions, economic analysis through payoff-optimizing computation does not provide a clear path for cooperation. We propose a new form of game–the ‘expected’ game–and illustrate its implications for theory and practice based on the prisoner’s dilemma example. We prove that cooperation can be a rational choice for players in reality in such games defined by a weighted set of payoffs of two or more different reference games.

## 1. Introduction

### 1.1 The expected prisoner’s dilemma, with rationally arising cooperation

The goal of economics as a science is to understand, explain and improve human-centered transactions, focusing on those of a commercial nature. So, any phenomenon that theoretically involves a Pareto inefficiency is a natural target of study; and, even more so when human subjects act so as not to experience that inefficiency. The *Prisoner’s Dilemma* game (PD) is one such phenomenon that has challenged economists. Defecting in the single-play (or even the low-repetition version) PD is dominant. In fact, there is *no* room for cooperation to arise (repeatedly or rationally) in such a phenomenon, yet it does when human subjects play it. Currently, there is *no* satisfying theoretical answer to that conundrum: A single mistaken choice should be severely punished [1]. A ‘crazy’ partner and a reasonable shadow of the future in a finitely-repeated game begs more questions about the idea and origin of the craziness itself [2]. And, the addition of external forces, like social-reputation effects, essentially removes the dilemma by altering the payoffs [3].

Our contribution is a gratifying response to the problem. We describe how cooperation *can* be a rational response to the PD by ‘modeling’ that game in a novel way. Instead of focusing on the choice of cooperation (C), we focus on the manufacture of the PD and how that can generate the rational play of C. We then discuss the implications of this new approach for game theory (and the related economics issues) more generally.

### 1.2. Manufacturing the ‘expected’ prisoner’s dilemma

**Fig 1:**
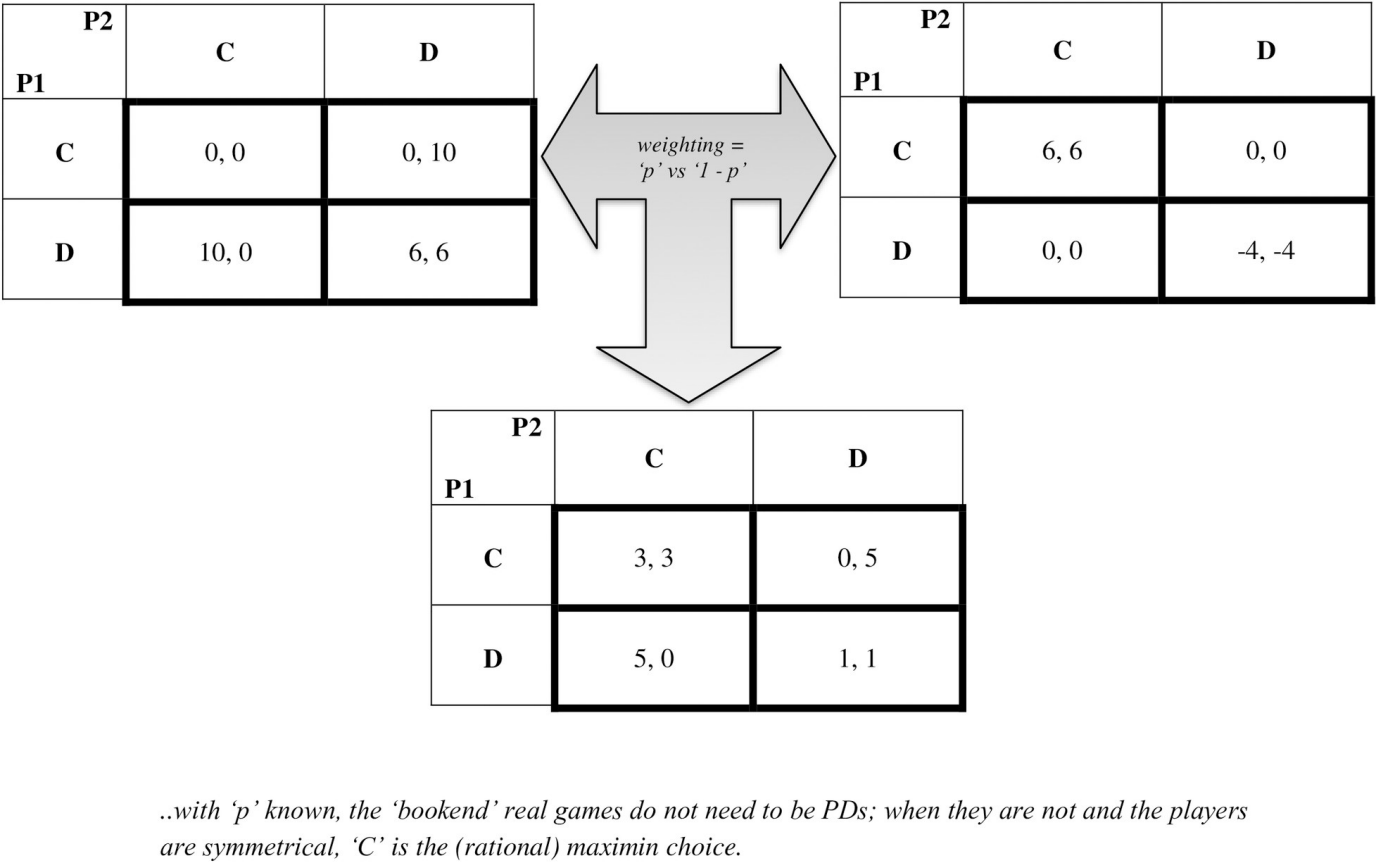
The ‘expected’ prisoner's dilemma.

An ‘expected’ game is manufactured when it is constructed from two (or more) *bookend games*–ones that involve the same players and the same possible actions–such that the probability-weighted payoffs in those bookends define the payoffs of that expected game. Fig 1 depicts this definition for symmetrical players and an expected PD game. Note that the expected game is not ‘real’ in the sense that any player believes that the final game actually played is the intermediate, expected one. More formally, each player faces two possible real versions of a game (e.g., depicting two alternative future contexts) against the same rival and with the same choices, but with differing payoffs. For simplicity, we also assume that each player knows the value of ‘p’–the probability of each version occurring–*prior* to choosing their actions. (Players choose actions simultaneously. Then a randomizer chooses the one bookend game that is played, based on ‘p’. Then the payoffs are determined based on the chosen actions and that one game. We assume that the game is accurately perceived by each player [theoretical or real] such that each player could calculate the expected game’s payoffs given 'p' and the bookend games' payoffs.)

Here, we focus on the interesting cases–those where the bookends are *not* PDs but the expected game is. These are the cases where the PD never actually occurs *ex post* in the real world but only *ex ante* in the expected decision-making world. These are the interesting cases because they provide insight into the possible construction of a ‘realistic’ version of a PD that isn’t actually realized, but only played in probability-weighted expectation. This is interesting because this is a novel version of the PD. This is interesting because such manufacturing of expected games is likely to exist in decision-making problems at a not-insignificant (but as yet to be studied) level. This is interesting because it provides the ability to manufacture such decision-problems, as well as the ability to deconstruct them and even possibly improve them. Besides being interesting, such cases are also important. The study of such cases is important because it provides: (i) a new way to understand the construction of [Pareto-inefficient] games–and, so, new ways to determine their origins and to affect them to improve the outcomes for at least one party; (ii) a new pathway for a ‘unique type of equilibrium’ [e.g., one which is stable through a specified range of ‘p’ or number of bookend games]; and, (iii) a new characterization of *ambiguity* defined by the case where ‘p’ is unknowable *ex ante* [in addition to its distribution, the distribution of that, and so on, also being *ex ante* unknowable].

We assert that the manufactured ‘expected’ game differs from existing seemingly-related concepts. It is *not* a variant of *incomplete information*, as no information asymmetry is involved (nor any accompanying dynamic deceptive signaling [4]). It is *not* a variant of a *Shapley problem*, as it is not focused on the value of any one coalition in a set of related games [5]. It is *not* a *Parrondo Paradox* [6], nor is it based on some other Brownian Ratchet [7] that improves the outcomes of expectedly ‘losing’ events by probabilistically cycling through an unbalanced game set; it is not based on dynamics or on cycling. And, it is *not* based on evolving games or on game transitions based on past play [8, 9]; it works in a one-shot decision involving unchanging bookend games. Further, we assert that, because of its uniqueness, it leads to a new and rational origin of C in a *de facto* PD.

#### 1.2.1 How cooperation arises in the expected prisoner’s dilemma

The existence of bookend games implies that the range and variance of the possible real payoffs from chosen actions in this model are significantly increased. And, that implication has an important impact on several standard objective functions, any one of which a (fully) rational player may wish to maximize through their choice of action in the model. In the example depicted in Fig 1, C is in the maximin choice. In fact, in our model, C can be the maximin or maximax choice, and can even be supported to be played with positive probability in order to maximize expected, risk-adjusted utility.

To be clear, in our model–where the expected game is anchored by bookend games–while some objective functions will not be affected (e.g., expected payoff and minimax-regret), others can be in a significant proportion of cases so as to entail C as being played with positive probability. This *only* can occur because the model increases the range and variance of real possible payoffs relative to the expected PD game (i.e., the game that defines the real possible payoffs in all other models).

#### 1.2.2 Simulation-based results

Consider the simplest representative case–the players are symmetric and ‘p’ = ½ (as we depict in Fig 1). It is relatively straightforward to run a Monte Carlo-type simulation to generate various possible bookend sets and then to analyze them to estimate the proportion of the population where C would be a rational choice for maximizing a standard objective function. Using an Excel spreadsheet, we run such a simulation where payoff perturbations to a given PD are randomly assigned to generate the two bookend games (here, where one bookend involves the positive and the other the negative perturbation). Thousands of such sets of bookends are generated and we remove all cases where at least one bookend is a PD itself to report our results.

We begin with the PD game depicted in Fig 1. Then we generate two bookend games by using the randomizer function to provide four payoff increments corresponding to the 4 different payoffs in the symmetric PD game; we set limits on the randomizer as -6 to +6, and we round the increments to three decimal places. We then use the randomizer function to generate four (0,1) flags, one for each of the four payoffs. To manufacture one bookend game we multiply the increment by the flag and add it to the original PD game’s payoff (doing so for all four payoffs). To manufacture the other bookend game we multiply the increment by the flag and subtract it from the original PD game’s payoff (doing so for all four payoffs). We generate 1000s of these bookend game sets and record them in a spreadsheet for analysis. We then analyze them in Excel based on the issue at hand. We identify which bookend sets have which strategy (C or D) as maximin and maximax, and then calculate their ratio in the population of interest. S1 Data File provide this analysis.

We calculate the proportion of these cases where C is maximin, maximax or one of either. When players are symmetric, we find that approximately 42% of the time C is maximin and 34% C is maximax (and roughly 69% C is at least one of the two). When the players are not symmetric, the more complex simulation finds that approximately 60% of the time C is maximin and 51% C is maximax (and roughly 86% C is at least one of the two). The bottom line is that *an expected PD can exist without any real ‘dilemma’* (i.e., Pareto-inefficiency), *where cooperation is a rational choice*.

The example from Fig 1 also provides a case where playing C with positive probability can be utility-maximizing for a sufficiently risk-averse player. Using the standard mean-variance-based utility function, and taken from the perspective of a player starting at the ‘usual’ equilibrium point [D, D], we can consider whether the player can unilaterally increase risk-adjusted utility by deviating from that point. If the player increases the proportion of playing C to one-tenth: the mean payoff decreases by 0.1 but the variance also decreases (by more than a magnitude more), such that if the constant absolute risk aversion coefficient is sufficiently high (e.g., on the order of 0.04, depending on the simulation of the draws [see S1 Data]), then this unilateral move to play C with positive probability is a rational choice.

To summarize, then, our new model–where an ‘expected’ game is manufactured from bookend games–provides new possibilities for rational players to choose previously-considered irrational actions. We have explained and exemplified how C can rationally arise in an expected PD as a maximin, or a maximax, or even a utility-maximizing choice.

### 1.3. Implications—To other games and other unknowns

This new definition of a manufactured ‘expected’ game and the results of this new PD analysis entail several interesting theoretical and practical implications. First, the analysis extends to *any* ‘dilemma-type’ (Pareto-inefficient) game, with *any* set of actions (with that relaxation on game possibilities making practical applications more likely). Further, the set of actions does not have to involve ‘cooperation’, and so the origination of *any* unpredicted (e.g., expectedly dominated) outcome may be newly studied in this approach. (This approach may also provide some new insight into where the ‘expanding’ part of an expected ‘expanding pie’ game originates, regarding at least one bookend’s payoffs.) Second, the construction of an ‘expected’ game yields a novel definition of ‘ambiguity’ to consider–that occurring when ‘p’ is unknown (and unknowable *ex ante*). Even with only two bookend games, the possible variety of distinct ‘expected’ games that can occur as ‘p’ alters in value can be as many as five. This makes it very difficult for any player to calculate a ‘best’ strategy. (Determining a best-programmed strategy, or one that is most-popular among human subjects, is left for future work.)

We provide no propositions regarding how human subjects will play this game, but some simple and testable ones would include: Human subjects play the 'expected PD' the same way they play the original PD [tested by having subjects play repeated rounds of each (single-shot and low-repetition) and comparing items like mean level of C chosen]; and, expected PD games involving bookend games with greater rational C-play produce greater levels of C-play in human subjects [tested by having subjects play expected PD games, some with no D-dominant bookends and some with, and comparing the mean level of C chosen].

Besides the implications for academic work in game theory, there are potential implications for practice. But, this depends on whether the expected game construction can be considered realistic. So, *is the expected PD something that could occur in business or nature*? A simple (symmetric) example (see Fig 1) has one bookend game with D dominant and the other C dominant (but could be any two separate actions). In the former, choosing C is synergistic, while other combinations are less rewarding (or penalizing, perhaps indicating disposal costs). In the latter, choosing D is synergistic, while other combinations are less rewarding or asymmetrically transferring to the player choosing D. If the two contexts are alternate futures where only one technological standard wins, in the latter case the better choice could indicate facing less rivalry when that standard (D) wins. The key to creating a realistic application is having at least two possible future contexts where the same set of actions exists but that pay off differently under each context in interesting, but justified ways. (This appears possible, at least to the current standard defined by the stories that are used to legitimize the PD, most of which do not actually exist [as cleanly as the payoffs indicate] in nature or in real business transactions.) As such, we look forward to future work on our new expected games.

## Supporting information

S1 Data (XLSX)

## References

[1] Axelrod R. (1984). *The Evolution of Cooperation*. Basic Books; New York.

[2] Kreps D. M., Milgrom P., Roberts J. & Wilson R. (1982). Rational cooperation in the finitely repeated prisoners' dilemma. *Journal of Economic theory*, 27(2), 245–252.

[3] Andreoni J. & Miller J.H. (1993). Rational cooperation in the finitely repeated prisoner's dilemma: Experimental evidence. *The Economic Journal*, 103(418), 570–585.

[4] Saloner G. (1987). Predation, mergers, and incomplete information. *The RAND Journal of Economics*, 18, 165–186.

[5] Shapley L.S. (1953). A value for n-person games. *Contributions to the Theory of Games*, 2(28), 307–317.

[6] Harmer G.P. & Abbott D. (1999). Losing strategies can win by Parrondo's paradox. *Nature*, 402(6764), 864–864.

[7] Lee Y., Allison A., Abbott D. & Stanley H.E. (2003). Minimal Brownian ratchet: an exactly solvable model. *Physical Review Letters*, 91(22), 220601 (1–4). 10.1103/PhysRevLett.91.220601 14683223

[8] Hilbe C., Šimsa Š., Chatterjee K. & Nowak M.A. (2018). Evolution of cooperation in stochastic games. *Nature*, 559(7713), 246–249. 10.1038/s41586-018-0277-x 29973718

[9] Su Q., McAvoy A., Wang L. & Nowak M.A. (2019). Evolutionary dynamics with game transitions. *Proceedings of the National Academy of Sciences*, 116(51), 25398–25404. 10.1073/pnas.1908936116 PMC6926053 31772008

